# Endophytes Increased Fruit Quality with Higher Soluble Sugar Production in Honeycrisp Apple (*Malus pumila*)

**DOI:** 10.3390/microorganisms8050699

**Published:** 2020-05-10

**Authors:** Hyungmin Rho, Victor Van Epps, Soo-Hyung Kim, Sharon L. Doty

**Affiliations:** 1 School of Environmental and Forest Sciences, College of the Environment, University of Washington, Seattle, WA 98195-2100, USA; tony0822@uw.edu or soohkim@uw.edu (S.-H.K.); 2 Department of Biology, University of Washington, Seattle, WA 98195-1800, USA; vaneppskimlab@gmail.com

**Keywords:** endophytes, plant growth-promoting symbionts, fruiting, phytohormone

## Abstract

Endophytes are fungi, bacteria, or yeast symbionts that live in the intercellular spaces or vascular tissues of host plants. Investigations indicate that endophytes isolated from the Salicaceae family (*Populus* and *Salix*) hosts provide several benefits that promote plant growth, including but not limited to di-nitrogen fixation, plant hormone production, nutrient acquisition, stress tolerance, and defense against phytopathogens. In exchange, the microorganisms receive domicile and photosynthates. Considering the known characteristics of nitrogen fixation and plant hormone production, we hypothesized that apple trees grown under nitrogen-limited conditions would show improved biometrics with endophyte inoculation. Our research objectives were to investigate the endophyte effects on plant physiology and fruiting. We examined these effects through ecophysiology metrics involving rates of photosynthesis, stomatal conductance and density, transpiration, biomass accretion, chlorophyll content and fluorescence, and fruit soluble sugar content and biomass. Our results showed evidence of the endophytes’ colonization in apple trees, decreased stomatal density, delayed leaf senescence, and increased lateral root biomass with endophytes. A highlight of the findings was a significant increase in both fruit soluble sugar content and biomass. Future research into the mechanistic underpinnings of this phenomenon stands to offer novel insights on how microbiota may alter carbohydrate metabolism under nitrogen-deficient conditions.

## 1. Introduction

For decades agricultural practices have treated crops with increased chemical usage for both greater yields and pathogen control [1]. The results have created short-term efficiency in food production. However, we presently face a shifting era. Soils bereft of nutrients and oversaturated with chemical applications are now a threat to food security for an ever-growing human population [2]. Moreover, the process has left behind severe environmental consequences such as aquatic hypoxic zones, contaminated ground drinking water, and heavy dependence on petrochemicals [1,3]. Maintaining crop production that will defend environmental integrity requires a deeper understanding of plant physiology, ecosystem dynamics, evolutionary mutualisms, and novel applications capable of integrating our understandings while maximizing crop potential. Bioprospecting of symbiotic microorganisms may offer such a possibility.

Endophytes are defined as free-living microorganisms that live in the intercellular spaces or vascular tissues of plants for at least part of their life cycle without harming their host [4,5,6,7]. They generally are bacterial, fungal, or yeast in nature, and research demonstrates they have a wide array of plant growth-promoting properties (PGP) [4,8,9]. Previously quantified PGP characteristics include but are not limited to biological di-nitrogen (N_2_) fixation, plant hormone production, nutrient acquisition, conferment of stress tolerance, immunity support, and more recently increased water-use efficiency of the host [7,10,11,12,13]. In exchange for their support, they receive photosynthates and a habitat with reduced microbial competitions [4,8,9].

Ongoing research continues to corroborate endophyte-conferred increases in plant biomass production under lowered or limited nitrogen (N) conditions [14,15,16,17,18,19,20,21,22]. This has triggered a rush of research looking into potential biostimulant applications, yet there has been little information regarding how endophytes may affect crop fruiting. Presumably, endophytes would improve fruiting as an extension of biomass production. While the host growth promotion benefits are known, many questions arise concerning endophytes as an excessive sugar sink, potentially detracting from fruit production. Some research reports endophytes as potential pathogens under varied environmental factors or life stages [9,23,24]. Endophytes have the capacity to demand resources too much from their host. Similarly, research also finds that fungal endophytes can modify the taste of wine fruit by altering their secondary metabolites [25]. The commercial fruit industry adheres to stringent guidelines for fruit quality. Market-ready fruit must be at certain levels of total soluble sugar content, fruit mass, water content, and palatability [26]. Consequently, apple crops are one of the most massive consumers of nitrogen/nutrients, irrigation, and pest management [27]. Based on this, we questioned whether endophytes would alter fruit viability and growth parameters of sweet, fleshy crops.

To test this hypothesis, we chose Honeycrisp (*Malus pumila* “Honeycrisp”) for our study. Apples are a $4 billion industry in the USA with a $15 billion downstream economy and over 130,308 ha of land dedicated to their production [28]. Honeycrisp apples are a newly favored market apple. Honeycrisp sales went from 94,000 boxes in 2007 to 3.7 million by 2011, and they are now the most produced apples in Washington State [29]. Honeycrisp apples are large, high water content apples averaging around 250 g apiece [30], demanding up to $8.83 per kg [29]. Their prized size and cost also mean, and perhaps more than other varieties, that consumers expect quality adherence. Honeycrisp apples require substantial inputs a year to uphold salable fruit cropping [31]. Mature apple trees need ≈67.3–100.9 kg of N/ha per year for growth and maintenance, although the exact amount of which is orchard life stage and area dependent [32]. Because of these factors, Honeycrisp apples made an ideal test crop for this study.

For this study, we grew 2-year-old trees for two years under nutrient-limited field conditions to test the maximized tree reliance on endophytic mutualism. We used an endophyte consortium comprising of nine endophytes with a broad platform of plant support, previously characterized and developed for optimum PGP traits including N_2_ fixation and phytohormone production [8,33,34,35,36,37,38]. We hypothesized that our inoculated Honeycrisp apple fruit trees would take up the consortia and establish a mutualistic relationship and would show improved rates of gas exchange and, therefore, carbon (C) assimilate leading to greater sugar content, water content, and overall biomass including fruit.

## 2. Materials and Methods

This experiment was conducted from April 2015 to March 2017 at the Douglas Research Conservatory at the University of Washington (47°39ʹ27ʺN, 122°17ʹ21ʺW; 10 m elevation above sea level), Seattle, WA, USA. The United States Department of Agriculture Plant Hardiness Zone of the study site is zone 8b (average annual extreme minimum temperature of −9.4 to −6.6 °C) [39].

### 2.1. Plant Materials and Growth Environment

Thirty 2-year-old Honeycrisp (*Malus pumila* “Honeycrisp”) semi-dwarf saplings on M106 rootstocks (newLeaf brand, Matz & Son Nursery, Portland, OR, USA) were purchased from a local nursery in April 2015. Trees were grown in an outdoor nursery in 37.8-L plastic nursery pots with nutrient-limited growth media (Sunshine Mix #2, Sun Gro Horticulture, Agawam, MA, USA). Trees were grown for one year to adapt to the growth environment. During the following 2016 experiment season from June to October, air temperatures averaged 12.6 °C, peaking at 19.6 °C during August. Average relative humidity (RH) was 70.4%. Average solar radiation was 5.06 MJ m^−2^ per day over the growing season, with the highest average during June at 5.74 MJ m^−2^ per day. Treatment replicates were grown in 10 pots × 3 rows with a randomized, weekly rotated placement of the pots. All trees were randomly placed and rotated once every two weeks. All trees received a 1-L weekly allotment of half-strength standard Hoagland solution [40] and were provided water to saturation as the growth environment dictated. We did not practice pruning or thinning of the trees over the course of the study as the plants were young trees. 

### 2.2. Endophyte Acquisition, Inoculation, and Confirmation

We acquired endophyte isolates from our previous work in 2005 and 2009. These strains were stored at –80 °C before use. We inoculated the potted apple trees in July 2016 as described below. We confirmed the colonization of the endophytes in apple tree tissue in March 2017.

A consortium of nine different endophytes was used in this experiment. The in vitro characteristics of the microbes were identified and quantified in previous publications [8,33,38]. Endophyte strains WP1, WP5, WP9, WP19, WPB, WW5, WW6, and WW7 were extracted from wild black cottonwood (*Populus trichocarpa*) and wild willow trees (*Sitka sitchensis*) sampled from their native habitat, Snoqualmie River, Western Washington, USA [8]. Strain PTD1, however, was isolated from hybrid poplar (*P. trichocarpa* × *P. deltoids*) [33]. All endophytes used in this experiment show a platform of plant growth-promoting properties including biological N_2_ fixation, plant hormone production, and nutrient acquisition. Atmospheric N_2_ fixation was previously confirmed with genetic testing for the nitrogenase reductase *nifH* marker gene [8,33,41]. Details of the endophytes are provided below in Table 1.

The selected nine endophytes were grown from single colonies individually in nitrogen-limited combined C liquid cell suspension media (NL-CCM) [42] for a 72-hour growth cycle. The optical density of the bacterial culture was measured using a spectrophotometer (UV-1700, Shimazu America Inc., Columbia, MD, USA). The nine strains were subsequently combined with N-free Hoagland’s media, creating a final inoculation solution with a standardized concentration of OD_600_ = 0.1 (equivalent to 1 × 10^7^ cells mL^−1^). A mock inoculum for the control group was prepared using only the N-free solution [8]. All microbiological tasks were done under aseptic, sterile conditions. One L of the inoculum was delivered to 15 treatment group trees, whereas 1 L of the mock inoculum was delivered to the other 15 control group trees on July 10, 2016. One L of the liquid inoculum and mock inoculum was poured into the plastic pots around the root boundaries of the plants. 

At destructive harvest on March 5, 2017 (238 days after inoculation, DAI), stem tissues that had a few leaf buds were tested for colony-forming unit (CFU) counts. Six plants per treatment were randomly chosen. Tissue surfaces were washed with dilute dish detergent prior to sterilization to remove any excess dirt. Plant tissues were sterilized with an 8-minute incubation of 3% NaOCl followed by four rinses with sterile de-ionized H_2_O. Samples from the final de-ionized H_2_O rinse were plated to ensure proper surface sterilization. Approximately 100 mg of sterilized tissue was masticated in 1.5-mL microtubes containing 400 μL NL-CCM solution with microtube pestles. The resultant solution was plated onto NL-CCM containing Petri dishes, and then after 72 h of incubation at room temperature, photos of the plates were taken on a photo stand. Colony-forming unit counts were processed with ImageJ software [43] to compare the bacterial counts in the colonized tissues between the treatment groups.

### 2.3. Simultaneous Gas Exchange and Chlorophyll Fluorescence Measurements

Gas exchange measurements were taken after fruit harvest on September 26, 2016 (60 DAI) using portable photosynthesis systems equipped with infrared gas analyzers (LI6400XT, LI-COR Inc., Lincoln, NE, USA). The measurements were taken on the fully expanded youngest leaves between 8 a.m. and 2 p.m. Two-cm^2^ leaf chamber fluorometers (6400-40, LI-COR Inc.) were set to measure gas exchange and chlorophyll measurements simultaneously. The measured parameters included net CO_2_ assimilation rate (*A*), intercellular CO_2_ concentration (*C*_i_), stomatal conductance (*g*_s_), transpiration rate (*E*), intrinsic water-use efficiency (iWUE) (calculated as *A*/*g*_s_), extrinsic water-use efficiency (eWUE) (calculated as *A*/*E*), electron transport rate (ETR), photochemical quenching (qP), and non-photochemical quenching (qN).

Settings of the sensor heads were 1500 μmol m^−2^ s^−1^ photosynthetic photon flux density (PPFD) for saturating light intensity, 400 ppm of CO_2_ concentration in the reference cell of the instruments, 20 °C block temperature, 300 μmol m^−2^ s^−1^ flow rate, and 40–70% RH in order to optimize the microclimate for photosynthesis during the measurements.

With the microclimate settings in the sensor heads, CO_2_ responses of photosynthesis (*A*/*C*_i_ curves) were recorded. A range of 400, 300, 150, 50, 0, 400, 400, 600, 800, 1000, and 1200 ppm of CO_2_ concentration in the reference cell of the instruments was used to construct the response curves. Measurements were taken after machine equilibrium was established for 1–2 min.

### 2.4. Stomatal Density Measurements

Leaf imprint samples were taken for stomatal development observation. Sample imprints were made on September 26, 2016 (60 DAI) with a standard process using clear nail varnish on the abaxial side of the fully expanded youngest leaves [44]. Three fields of view were photographed, stomata summed, and averaged. ImageJ program was used with an add-on package to count the numbers of stomata of the specimens with 40× magnification from a standard compound microscope [43].

### 2.5. Chlorophyll Content and Fluorescence Measurements

Plant chlorophyll content was measured beginning on August 29, 2016 (50 DAI) at circa 10-day intervals until senescence (90 DAI). Indirect in vivo chlorophyll content (SPAD) was measured on leaves of the same branch three times and averaged. A handheld chlorophyll meter (SPAD-502, Konica-Minolta, Japan) was used in all recorded SPAD measurements.

Leaf chlorophyll fluorescence was measured twice during the experiment. The first was taken on August 30, 2016 (51 DAI), and the second was taken on October 6, 2016 (88 DAI) during the onset of senescence. Measurements were observed before sunrise with dark-adaptation cuvettes applied on the fully expanded youngest leaves at sunset the previous day and overnight. All measurements were taken with a handheld field fluorometer (OS-30p+, Opti-Sciences, Hudson, NH, USA). Maximal photochemical efficiency of photosystem II (F_v_/F_m_) was recorded and used to assess the health status of the plants under N-limited conditions.

### 2.6. Growth, Fruit Metrics, and Destructive Biomass Allocation Measurements

After receipt and before transplanting into pots, we measured shoot, root, and total length and total fresh weight of the apple trees for initial data collection of growth on April 12, 2015. We repeated the same growth measurements at destructive harvest on March 5, 2017 to collect final data. The growth over the growing seasons 2015–2016 was evaluated by calculating the increments of the measured growth parameters. 

All fruits were harvested on September 10, 2016 (62 DAI), weighed for fresh mass, total soluble sugar content sampled, and kiln-dried (at 70 °C for constant weights) for the subsequent dry mass measure. Total soluble sugar content was measured by pressing a small sliver of fruit in a hydraulic plant sap press (Plant Sap Press#2720, Spectrum Technologies Inc., Aurora, IL, USA). Approximately 100 μL samples was measured using a digital total soluble sugar refractometer (HI 96803, Hannah Instruments, Woonsocket, RI, USA).

Trees were harvested on March 5, 2017 (238 DAI) during winter dormancy and measured for shoot, root, trunk, and fresh/dry mass growth. Due to variability in rootstock size, aboveground biomass was taken at graft and roots were removed at rootstock.

### 2.7. Statistical Analysis

All physiological parameters were statistically analyzed using an unpaired two-sample *t*-test with R v.3.5.1 [45]. The normality of the distribution was checked and confirmed before running the *t*-test. The sample size of the measured variables varied depending on the type of measurements, but for each statistical comparison, we used the same sample size for the mock-inoculated control and endophyte inoculated each.

## 3. Results

### 3.1. Colony-Forming Unit Counts

**Figure 1:**
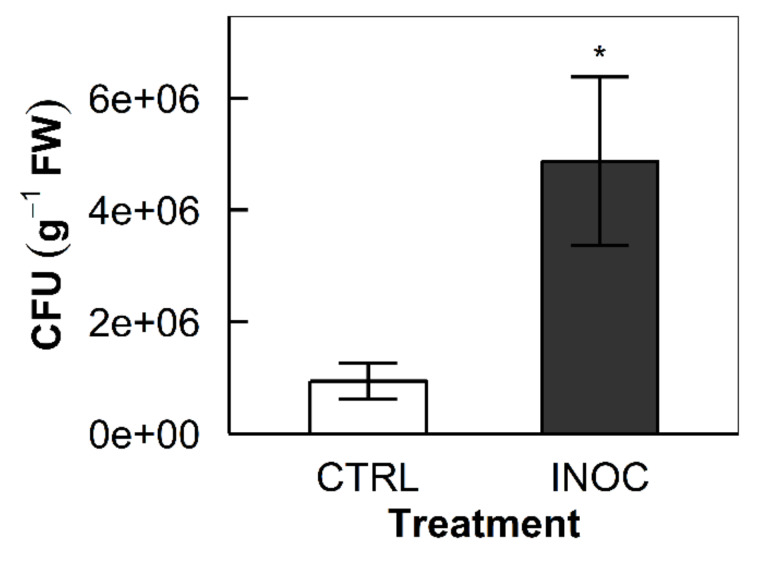
Colony-forming unit (CFU) counts of the apple tree tissue extracts: Bars indicate the means of the replicated samples from each treatment group (*n* = 6, CTRL and INOC for mock-inoculated control and endophyte-inoculated group, respectively). Error bars represent the standard errors of the means (* *p* = 0.047).

The inoculation of the endophytes increased CFU counts of the apple tree tissue extracts on the selective NL-CCM media, confirming endophyte colonization. Colony-forming unit counts from bud and stem extractions counted 9.40 × 10^5^ and 4.87 × 10^6^ CFU g^−1^ fresh plant tissue for the control and the inoculated samples, respectively (*p* = 0.047, Figure 1). No bacteria were observed for the rinse water plates from both control and inoculated samples.

### 3.2. Leaf Physiological Traits

**Figure 2:**
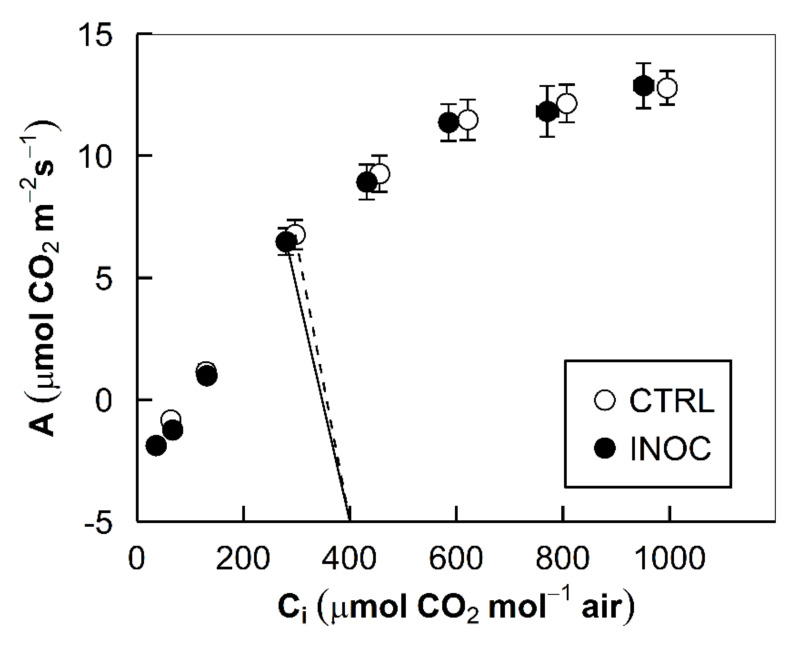
CO_2_ response curves of the apple tree leaves: Photosynthetic net CO_2_ assimilation rates (*A*) are plotted over a range of intercellular CO_2_ concentrations (*C*_i_) of the leaves. Points indicate the means of the replicated samples at each pair of *A*/*C*_i_ from each treatment group (*n* = 15, CTRL and INOC for mock-inoculated control and endophyte-inoculated groups, respectively). Error bars represent the standard errors of the means. Lines indicate the values corresponding to the responses under the ambient CO_2_ conditions (circa 400 ppm).

The inoculation of the endophytes did not change photosynthetic and transpirational traits of the apple trees on the leaf level at the ambient CO_2_ level of 400 ppm. Photosynthetic net CO_2_ assimilation rate (*A*) measured 6.768 and 6.488 μmol CO_2_ m^−2^ s^−1^ for the control and the inoculated groups, respectively (*p* = 0.725, Figure 2 and Table 2). Stomatal conductance (*g*_s_) measured 0.126 and 0.101 mol H_2_O m^−2^ s^−1^ for the control and the inoculated groups, respectively (*p* = 0.110, Table 2). Transpiration rate (*E*) measured 1.545 and 1.273 mmol H_2_O m^−2^ s^−1^ for the control and the inoculated groups, respectively (*p* = 0.113, Table 2). Water-use efficiency showed opposite trends to *g*_s_ and *E* as slightly higher iWUE and eWUE were observed for the inoculated group, but they were not statistically significant (*p* = 0.113, and 0.095, respectively, Table 2).

**Figure 3:**
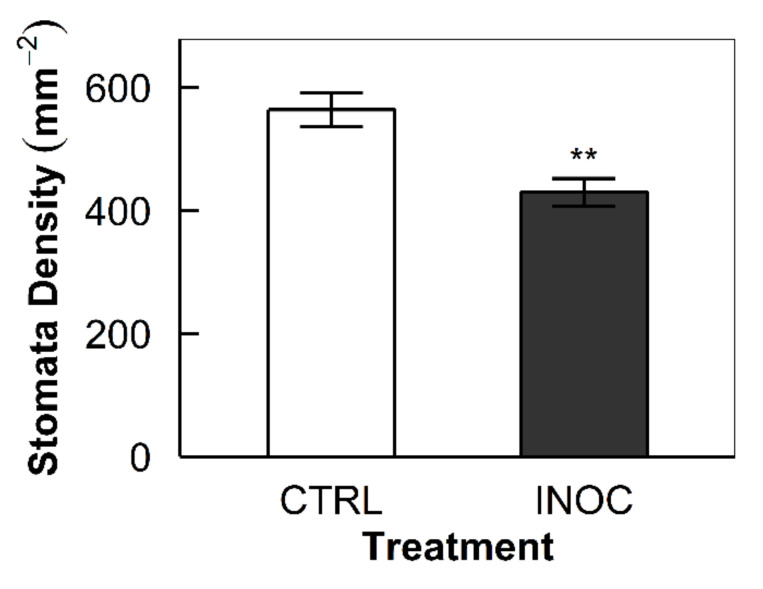
Stomata density of the apple tree leaves: Bars indicate the means of the replicated samples from each treatment group (*n* = 8, CTRL and INOC for mock-inoculated control and endophyte-inoculated group, respectively). Error bars represent the standard errors of the means (** *p* = 0.001).

The inoculation of the endophytes reduced the number of stomata developed on the leaf surface of the apple trees. Stomata density measured 564.3 and 430.2 each mm^−2^ for the control and the inoculated group, respectively (*p* = 0.001, Figure 3).

**Figure 4:**
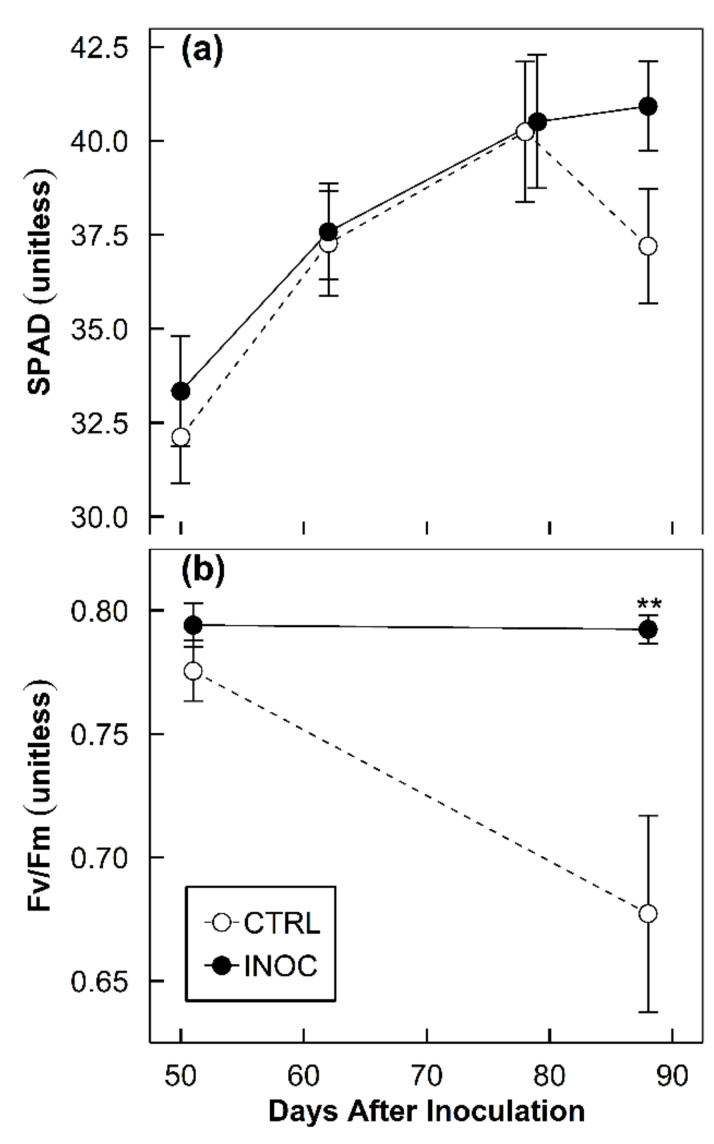
Time course responses of chlorophyll characteristics of the apple tree leaves: (**a**) chlorophyll content; (**b**) chlorophyll fluorescence. Points indicate the means of the replicated samples from each treatment group (*n* = 13-15, CTRL and INOC for mock-inoculated control and endophyte-inoculated group, respectively). Error bars represent the standard errors of the means (** *p* = 0.001).

The inoculation of the endophytes did not change overall seasonal chlorophyll content but delayed the decrease at senescence. Seasonal SPAD measured 36.8 and 37.7 for the control and the inoculated groups, respectively (*p* = 0.410). However, the data measuring 88 DAI showed a difference between the control and the inoculated. For the control group, SPAD measured 37.2 and 40.9 for the inoculated group (*p* = 0.051, Figure 4a). The inoculation of the endophytes increased the overall maximum photochemical efficiency of photosystem II on the leaves of the apple trees. Seasonal F_v_/F_m_ for the control group measured 0.723 and 0.793 for the inoculated group (*p* = 0.007, Figure 4b). Fifty-three DAI F_v_/F_m_ for the control group measured 0.776 and 0.794 for the inoculated group (*p* = 0.023, Figure 4b). Eighty-eight DAI F_v_/F_m_ for the control group measured 0.677 and 0.792 for the inoculated group (*p* = 0.012, Figure 4b). 

### 3.3. Biomass Allocation and Fruit Metrics

The endophyte-inoculated apple trees showed greater trunk width, root, and total dry weight at harvest compared with those of the control trees. The other biomass parameters measured were found similar between the control and the inoculated trees. The fruits from the inoculated apple trees had greater fresh weight and higher total soluble sugar content compared with those of the controls. Destructive harvest fresh trunk width for the control group measured 1.84 and 1.95 cm for the inoculated group (*p* = 0.038, Table 3). Destructive harvest fresh weight growth for the control group measured 0.796 and 0.842 kg for the inoculated group (*p* = 0.560, Table 3). Destructive harvest dry weight for the control group shoots measured 0.164 and 0.174 kg for the inoculated group (*p* = 0.240, Table 3). Destructive harvest dry weight for the control group roots measured 0.107 and 0.142 kg for the inoculated group (*p* = 0.028, Table 3). Total dry weight for the control group measured 0.271 and 0.315 kg for the inoculated group (*p* = 0.022, Table 3). Total shoot growth for the control group measured 0.158 and 0.231 m for the inoculated group (*p* = 0.310, Table 3). Total root growth for the control group measured 0.530 and 0.428 m for the inoculated group (*p* = 0.150, Table 3). Fresh and dry weight of harvested fruit per tree was lower in the inoculated group, but the differences were not significant (*p* = 0.216 and 0.189, respectively, Table 4). Per-fruit fresh weight of harvested fruit for the control group measured 144.4 and 165.2 g for the inoculated group (*p* = 0.008, Table 4). Per-fruit dry weight of harvested fruit for the control group measured 27.7 and 31.0 g for the inoculated group (*p* = 0.07, Table 4). Total soluble sugar content of harvested fruit measured 14.0 and 15.4° Brix for the control and the inoculated group, respectively (*p* < 0.001, Table 4).

## 4. Discussion

### 4.1. Endophyte Symbiosis Established

To confirm the observed physiology and biomass data in this study was associated with the inoculation of the endophytes, verification of the colonization of the endophytes in planta was required. As such, we performed a selective-CFU extraction and count on control and inoculated group plants and compared the CFU of those. Consistent with previously published protocols on apple trees, we extracted from woody stem and leaf buds during dormancy [46]. Initial extraction to test our protocols showed an 8-minute incubation worked best for our test subject and substrates. Our final extraction confirms positive and significant inoculation. Control trees averaged 9.40 × 10^5^ CFU g^−1^ fresh plant tissue, whereas inoculated trees counted 4.87 × 10^6^ CFU g^−1^ fresh plant tissue (*p* = 0.047, Figure 1). The CFU count of the inoculated trees is comparable to the level previously reported in rice (*Oryza sativa*) shoots with the same endophyte consortium [47]. All plated rinse waters from incubation were clear of surface contamination corroborating the CFUs as endophytic in origin. The results show a clear difference in microbial populations between test groups that may be due to the inoculated strains. Furthermore, because we tested during dormancy, we demonstrate that the established mutualisms are apt to carry over into the following growing season. Future genetic validation of CFU extractions and/or the green fluorescent protein tagging can capture a better picture of which bacteria isolates colonize where in apple trees and how they negotiate growing season into dormancy. 

### 4.2. No Changes to Gas Exchange Properties with Endophytes

Overall, the results in this study aligned with both the hypotheses of the study and previous results [36,37,41,47]. The trends in photosynthesis (*A*) and *A*/*C*_i_ curves, however, did not match our expectations. No statistical difference was found in *A* measured at the ambient CO_2_ condition (400 ppm) between the control and the inoculated trees (*p* = 0.725, Table 2). Rates were measured post fruit harvest and were not significant. This result confers with previous findings where significant differences in assimilation were not reported [37]. Two reasons may be underlain. First, measurements were taken post fruit production. Plants have higher rates of photosynthesis during fruit production [48]. It is plausible that, based on the fruit and WUE (water-use efficiency) data (see below), there was a more remarkable difference during this time. Second, it is hypothesized from previous findings that endophytes provide the plant with available photosynthetic C substrate through respiration [49]. This means the data may not reflect actual rates of assimilation. 

Stomatal conductance and transpiration did not follow anticipated patterns. Average *g*_s_ in our inoculated trees was 0.101 versus 0.126 mol H_2_O m^−2^ s^−1^ in our control group (*p* = 0.110, Table 2). Again, with transpiration, there were no differences. Average *E*s in our inoculated trees were 1.273 versus 1.545 mmol H_2_O m^−2^ s^−1^ in our control group (*p* = 0.113, Table 2). Decreased *g*_s_ *E* and increased WUE in the endophyte-inoculated under N-limited conditions were observed in our previous results in poplar clone cuttings [36] and in rice [50,51]. Similar trends but with *p*-values slightly over the marginal significance level of 0.10 may indicate that the effects of endophytes on the gas exchange parameters in apple trees are low and that the sample size in replication was not enough to detect the statistical significance at the 90% confidence level. Given that, future studies would be required to be conducted with a larger sample size. Another reason for the slight differences may have come from measurements taken earlier in the day with trees transferred from the field to the climate-controlled head house at the research center. Under less diurnal stress, the effect of the endophytes may be muted. However, the data in this study confer with previous experiments with corn (*Zea mays*) and low N conditions [41]. In this research, significant differences in *A*, *E*, and *g*_s_ were reported only in high N conditions. Based on the data from this study and previous research, it follows that our inoculated group’s N levels were likely too low to present significant differences in *A*, *E*, and *g*_s_. Although future research is needed, these trends in both previous results and here reported seem to hold greater potential under typical growing conditions. 

### 4.3. Reduced Stomata Density with Endophytes

When considering the hypothesis that endophytes offer intercellular CO_2_, secondary questions arise as to how this may, if at all, impact stomata density. If there were added C available from internal endophytic respiration, then hypothetically, the inoculated plants would need fewer stoma to match their C needs [49]. Producing, maintaining, and operating stomata is costly, and therefore, it would prove beneficial to have fewer while maintaining adequate C balance. We took abaxial side leaf imprints to look at the prevalence of stomata between treatment groups. Inoculated trees had an average of 430.4 compared with the control group, which had an average of 564.3 stoma mm^−2^ (Figure 3). This finding between the two was significantly different and upheld our hypothesis (*p* = 0.001). This result also aligns with previous findings with research involving rice [50]. Although the data are compelling, lacquer imprints can be highly variable in quality and surety despite consistency in the method. Genomic confirmation of the presented findings will be an important link in our ecophysiological understandings.

### 4.4. Delayed Senescence with Endophytes

Chlorophyll content measured as SPAD is commonly used to track N as a proxy [52] (p. 276). Chlorophyll requires N for its formation, and therefore, plants with low N will show yellowing in the leaves [52] (p. 352). Our tested endophytes in this research are diazotrophic (Table 1). If the microbes are providing additional N under the N-limited conditions, inoculated plants should show higher chlorophyll content over control plants. The chlorophyll content data positively reflect this in part. Over season mean chlorophyll content for the control plants was 36.8 compared with 37.7 for the inoculated (*p* = 0.410, Figure 4a). Over season findings were not significant but looking into individual days of readings reveals interesting trends. On 79–82 DAI, we saw SPAD values for the control plants spiking above inoculated trees followed by a rapid drop below the inoculated plants (Figure 4a). Our final SPAD measurement showed a mean 37.2 for the control subjects versus 40 for the inoculated group. These measurements were significant to the 90% confidence range (*p* = 0.065, Figure 4a). Data points 79–82 DAI and 89 DAI were taken during the final week of September and the first week of October. The spike and subsequent drop in SPAD in the control group point to an earlier senescence onset in contrast to the inoculated ones. Nitrogen mobilization at the start of senescence is well documented, and we posit this as the source of the observed phenomena [52,53] (p. 338). The SPAD data, despite this, does not entirely match other researches previously conducted. Preceding experiments report significant differences in SPAD between control groups and those inoculated with consortia in poplar and maize [37,41]. Perhaps a factor in the differing outcomes of this study was due to field settings of the experiment and SPAD protocols. Future studies using endophytes under nutrient limitations in field conditions should account for these discrepancies. 

Notwithstanding potential SPAD measuring discrepancies, the chlorophyll fluorescence data supports and confirms the SPAD data. Seasonal data bore significant mean F_v_/F_m_ differences for the control plants at 0.723 compared with the inoculated at 0.793 (*p* = 0.007). Interestingly, F_v_/F_m_ was not significantly different at the end of August. At this time, control trees measured a mean F_v_/F_m_ of 0.776 versus inoculated trees, which measured a mean 0.794 (*p* = 0.230, Figure 4b). Measurements following these at the end of the season in October relayed a different story. Control trees measured a mean F_v_/F_m_ of 0.677 versus inoculated trees, which measured a mean 0.792 (*p* = 0.012, Figure 4b). End-of-the-season measurements were statistically significant, indicating that control trees were in senescence while the inoculated remained active and functioning as the SPAD data also suggested [53,54]. Moreover, inoculated trees never fell below 0.790 of F_v_/F_m_, which is considered the cutoff for healthy plants [54]. This is remarkable considering the low N conditions and reflects effectual N fixation provided by the diazotrophs. This delay in senescence was another plausible cause for greater allocation of carbohydrates in the inoculated fruits.

Both the SPAD and the F_v_/F_m_ data point to slowed senescence in inoculated plants. Perhaps the extra N, hormones, and nutrient acquisition make a longer growing season more attainable for the trees. Conversely, perhaps ongoing seasonal photosynthesis maintains endophytic health and improves carbohydrate storage for overwintering. Hypothetically this is beneficial for both host and mutualists. The mechanisms underlying this are not known, but it may be related to 1-aminocyclopropane-1-carboxylic acid (ACC) deaminase [55,56]. 

The plant hormone ethylene is involved throughout plant development, including response to stress, leaf senescence, and leaf abscission [56]. Increased levels of ethylene are seen in response to drought, ultraviolet damage, extreme heat, etc. [57]. To respond to such stress, the plant must upregulate the production of ACC, the precursor to ethylene [56,58]. This type of plant stress-hormone response can slow plant growth and can have deleterious consequences in plant health [56]. It is argued that a primary characteristic of plant growth-promoting endophytes is the endophytic production of ACC deaminase enzymes [59]. The deaminase cleaves ACC to form ammonia and ketobutyrate, not only providing what some argue suppression of deleterious stress hormone but also possibly freeing up N to be recycled [56]. If ACC deaminase cleaves ethylene in response to stress, then it may also stave off the onset of senescence. Ali et al. [60] placed carnation flower cuttings in a solution with endophytic bacteria known to produce ACC deaminase and tracked flower petal senescence. Flower senescence was delayed in the endophyte treatment group by 2–3 days [60]. We suggest a similar effect is responsible for the delayed senescence in our inoculated trees. Given some of our endophyte strains used in the present study can produce ACC deaminase [36] but given its reported prevalence amongst endophytes and the data here provided, this is a likely underlying cause. Future research is needed to further characterize our consortia and the potential benefit of delayed senescence on woody crops. 

### 4.5. Increased Trunk Width and Root Mass with Endophytes

Just prior to destructive harvest, trunk width was measured with precise, machined Starett calipers. Control trees averaged 1.84 cm wide compared to the greater width of inoculated trees at 1.95 cm. The widths were significantly different at the 95% confidence level (*p* = 0.038, Table 3). Cambium growth is subject to indole-3-acetic acid (IAA) production and regulation [61]. Greater cambium growth is yet another proxy for endophytic plant growth support through plant hormones. We propose that endophytic plant hormone production may influence not only primary growth biomass but also secondary in woody species. Long-term endophyte studies on secondary growth support will be an important area of study for a deeper understanding of this initial data.

Because of previous findings with improved WUE with endophyte inoculation, this research looked at fresh weight growth prior to dry harvest weights. Upon early inquiry into this data set, we found a significant difference between fresh weights between treatments, but this did not hold when we compared the fresh weights to the original fresh weight data. We found no difference in fresh weight growth between treatments with the control at 0.796 versus the inoculated at 0.842 kg (*p* = 0.560, Table 3). The trees were in full dormancy during this final fresh weight measurement and bud burst during the first. With ongoing data emerging, pointing to increased endophyte-induced WUE, future research data collection will benefit from improved fresh weight protocols.

Post destructive harvest and kiln-dried control tree shoots (secondary growth only) weighed a mean of 0.164 compared with inoculated trees at 0.174 kg. Despite the trunk width data, inoculated plants did not show improved shoot mass (*p* = 0.240, Table 3). Post destructive harvest and kiln-dried control tree roots (secondary growth only) weighed a mean of 0.107 kg compared with inoculated trees at 0.142 kg. Inoculated group data do reveal significantly increased root biomass (*p* = 0.028, Table 3). Control trees averaged 0.158 m of shoot growth in contrast to inoculated trees, which showed 0.231 m of growth. Control trees averaged 0.530 m of root growth in contrast to inoculated trees, which showed 0.428 m of growth. Again, shoot growth was greater with endophytes but not statistically (*p* = 0.310, Table 3). Root growth, conversely, showed greater growth in length, also not statistically so (*p* = 0.150, Table 3). The increased root dry weight and the less root growth in the inoculated trees together may suggest increases in lateral root growth rather than primary root growth. This lateral, denser root architecture could be attributable to hormonal responses triggered by the endophytes. Lateral root formation can be affected by phytohormone-producing PGP rhizobacteria [62].

The experimental results from this study did not entirely follow previous findings in other plant species such as maize, rice, and poplar. Although the patterns of growth and data trend do match, preceding publications reported more significant differences in growth parameters between treatments [37,41]. The presented data only show this for root mass, also in support of endophyte phytohormone support [63]. Uncontrolled field conditions, insufficient nutrient supplementation, smaller sample sizes, or the demands of sweet fruit production were possibly attributed to differences in the data collected in this study. Nonetheless, the treatment groups displayed expected growth patterns and greater secondary growth and root mass. Moreover, we saw an increase in control root length signifying root searches for more adequate nutrition, also upholding our expected controls’ response. Overall, the growth data from this pilot study provide some points for consideration for a longer study with woody species, especially considering endophyte effects on secondary growth.

### 4.6. Increased Fruit Metrics with Endophytes

The control trees provided more individual fruits than our inoculated (*n* = 59 vs 32). By design, we inoculated after the trees flowered and began fruit set. Because of this, we believe this occurrence is happenstance associated with the randomness of field conditions and nonmanual fertilization. No notable aborted fruits were logged, and care was taken not to knock apples off of their trees during measurements and routine care. All fruits were harvested on the same day: September 10, 2016, when the first fruits were observed to drop in the control group. Upon harvest, fresh FW per fruit of the treatment group fruit showed a significantly higher value over the control group by an average of 20.8 g (*p* = 0.008, Table 4). After drying the fruit, the inoculated fruits’ mean weight was still greater but only at the 90% confidence level (*p* = 0.070, Table 4). Total soluble sugar content was recorded for all fruit during harvest. Fruit from inoculated trees showed significantly higher total soluble sugar levels, 15.4 vs 14.0° Brix (*p* < 0.001, Table 4). Honeycrisp apples with a Brix value above 14 are considered excellent and commercially viable quality [64]. Of the 32 harvested apples from the inoculated treatment, only three fell below this standard whereas 19 (~33%) of the control group were below commercial standard. Although it was not quantified, fruits also displayed visually different appearances. Inoculated fruits were redder in coloration and appeared more mature and ripened. The higher fruit soluble sugar content and weight supports this observational data.

It is well documented that the combination of auxin (IAA), gibberellins (GA), and cytokinin (CK) are major players in fruit setting physiology [65,66,67]. All three hormones are increased at fruit set, but more specifically, the interplay of IAA and GA hormones are observed in fleshy fruit development [65,66]. Furthermore, IAA is notably responsible for growing fruits’ cell expansion, which, along with osmotic compounds such as sugars and organic acids, are primary factors in determining fruit size [66,68]. Boosted IAA is important throughout fruit development; however, it impedes fruit ripening and finishing. To accomplish this, IAA must be lowered, requiring cross talk with other hormones [65,66]. Previous research indicates that abscisic acid (ABA), brassinosteroids (BR) and ethylene are important fruit finishers, inducing ripening and color changes [67,69,70,71]. Other hormones such as jasmonic acids (JA) and salicylic acid (SA) are associated with the production of volatile acids that give fruit characteristic scents, signaling that the fruit is ready to eat and to attract would-be seed dispersers [69,72]. In sum, current research shows evidence for IAA, GA, CK, and BR at fruit set; IAA, GA, and BR at fruit growth; IAA and ABA at fruit maturation; and CK, BR, JA, and SA during fruit ripening (for further information not reviewed here; see Reference [65]). 

All endophytes used in this research were previously confirmed for the aforementioned hormones SA, JA, IAA, ABA, GA_3_, and Brs [36] (Table 1). These hormones are not only associated with plant growth but also fruit set, growth, maturation, and ripening. Hence, it stands to reason that the hormone production by our treatment endophytes not only promotes plant growth as previously reported but also appears to play an important role in improved fruiting. Ongoing research in the field of plant hormone-regulated fruiting physiology is gaining momentum with new genomic approaches. Little is known of many of the underpinnings of regulation, particularly considering the complexities of hormone cross talk and feedback nuances. That said, the role of microbes in this equation must also be considered. Research shows field sampling of various fruit crops to have numerous endophytes [46,73,74,75]. It seems probable that under growing conditions with healthy, bioactive soils, plants will recruit whatever microbes available in the area to aid in their health, growth, and fruiting. This raises many questions not only for the underlying mechanisms of these mutualisms but also for what to support them through consortia development and application programs. 

The potential hormonal effects alone are not likely to fully account for the increased cell expansion, water weight, and sugar content of the fruit. There must be an adequate substrate. Neither the rates of photosynthesis, conductance, nor transpiration data support the fruit data findings. Upon further inquiry into gas exchange measurements, we did, however, find an increased eWUE at a marginal level (Table 2, *p* = 0.095). This emerging data helps explain the occurrences of the improved fruit water content and growth while giving some information about how the gas exchange tools may not capture the precise levels of C assimilation performed when endophytes are present. More analysis of this emerging data is required to draw further inferences. 

## 5. Conclusions

Here, we provide the research to show that endophytes can increase sugar production, water content, and per-fruit-biomass in fleshy fruits. We also present evidence for endophyte-induced delayed senescence as an indicator of higher plant functioning and a likely source of increased carbohydrate source to sink. Despite their limited nutrition, inoculated trees were still able to outperform the control group with greater assimilation in fruit, root mass, and secondary growth. This pilot study provides a glimpse into the expanding base of endophyte knowledge. Our findings suggest that endophytes are implicated not only in host growth physiology but also in fruit maturation and ripening, although the impacts of the endophyte symbiosis on fruit set remains as an item of further investigation. These phenomena open important fields of biochemical, mechanistic, and evolutionary inquiry. With a better understanding of mechanistic underpinnings of endophyte symbiosis, we may be able to achieve advances in the application of the microbial technology into commercial agriculture in the face of modern food security and agriculture challenges.

## Figures and Tables

**Table 1:** Composition of the endophyte consortium used in the study.

Isolate^1^ Name	Closest rRNA Match	Reference
PTD1	*Rhizobium sp.*	[33,36]
WPB	*Burkholderia sp.*	[8,35,36]
WP19	*Acinetobacter sp.*	[8,36]
WP5	*Rahnella sp.*	[8,36]
WP9	*Burkholderia sp.*	[8,36]
WW5	*Sphingomonas sp.*	[8,36]
WW6	*Pseudomonas sp.*	[8,36]
WW7	*Curtobacterium sp.*	[8,36]
WP1	*Rhodotorula sp.*	[34,36]

^1^ Isolate phytohormone production (SA, salicylic acid; ABA, abscisic acid; IAA, indole-3-acetic acid; JA, jasmonic acid; GA_3_, gibberellins-3-acid; Brs, epibrassinolides) in growth cultures of endophytes.

**Table 2:** Descriptive and inferential statistics of leaf physiological trait parameters^1^ at the ambient CO_2_ level from the CO_2_ response curves (Figure 2): The means of responses are provided with the standard errors of the means in parentheses (CTRL, mock-inoculated control; INOC, endophyte-inoculated; *n* = 15). *p*-values of an unpaired two sample *t*-test results on each parameter are provided.

Treatment	*A*	*C*_i_	*g*_s_	*E*	iWUE	eWUE	ETR	qP	qN
	μmol CO_2_ m^−2^ s^−1^	μmol CO_2_ mol^−1^ air	mol H_2_O m^−2^ s^−1^	mmol H_2_O m^−2^ s^−1^	µmol CO_2_ mol^−1^ H_2_O	µmol CO_2_ mmol^−1^ H_2_O	μmol CO_2_ m^−2^ s^−1^	Unitless	Unitless
CTRL	6.768	296.9	0.126	1.545	56.37	4.496	57.00	0.306	1.812
	(0.605)	(8.590)	(0.009)	(0.106)	(5.197)	(0.354)	(5.181)	(0.027)	(0.088)
INOC	6.488	280.0	0.101	1.273	67.18	5.304	57.20	0.295	1.841
	(0.549)	(6.610)	(0.010)	(0.126)	(4.145)	(0.307)	(5.131)	(0.024)	(0.076)
*p*-value	0.725	0.128	0.110	0.113	0.113	0.095	0.976	0.782	0.804

^1^*A*, photosynthetic net CO_2_ assimilation rate; *C*_i_, intercellular CO_2_ concentration; *g*_s_, stomatal conductance; *E*, transpiration rate; iWUE, intrinsic water-use efficiency (calculated as *A*/*g*_s_); eWUE, extrinsic water-use efficiency (calculated as *A*/*E*); ETR, electron transport rate; qP, photochemical quenching; qN, non-photochemical quenching.

**Table 3:** Biomass characteristics of apple trees at harvest: The means of responses are provided with the standard errors of the means in parentheses (CTRL, mock-inoculated control; INOC, endophyte-inoculated; *n* = 15). *p*-values of an unpaired two sample *t*-test results on each parameter are provided with significance codes of *, **, and *** for <0.05, 0.01, and 0.001, respectively.

Treatment	Trunk Width	Shoot Length	Root Length	Total Length	Shoot Growth ^1^	Root Growth ^1^	Total Growth ^1^	Total FW ^2^	Total FW Growth ^1^	Shoot DW ^3^	Root DW ^3^	Total DW ^3^
	cm	m	m	m	m	m	m	kg	kg	kg	kg	kg
CTRL	1.840	1.477	0.806	2.284	0.158	0.530	0.694	1.164	0.796	0.164	0.107	0.271
	(0.033)	(0.027)	(0.034)	(0.039)	(0.018)	(0.040)	(0.048)	(0.078)	(0.066)	(0.005)	(0.007)	(0.010)
INOC	1.946	1.477	0.804	2.282	0.231	0.428	0.605	1.259	0.842	0.174	0.142	0.315
	(0.035)	(0.027)	(0.045)	(0.048)	(0.066)	(0.055)	(0.062)	(0.052)	(0.041)	(0.005)	(0.013)	(0.014)
*p*-value	0.038*	0.997	0.966	0.971	0.310	0.150	0.271	0.321	0.560	0.240	0.028*	0.022*

^1^ Nondestructive growth was determined by subtracting the initial 2015 records from the final 2017 records of the measured parameters. ^2^ FW, fresh weight of the samples. ^3^ DW, dry weight of the samples.

**Table 4:** Apple fruit characteristics (*n* = 59 and 32 for CTRL and INOC, respectively): The means of responses are provided with the standard errors of the means in parentheses (CTRL, mock-inoculated control; INOC, endophyte-inoculated; *n* = 15). *p*-values of an unpaired two sample *t*-test results on each parameter are provided with significance codes of *, **, and *** for <0.05, 0.01, and 0.001, respectively.

Treatment	Fruit Count per Tree	Total Soluble Sugar Content	Fruit FW ^1^ per Tree	Fruit DW ^2^ per Tree	Fruit FW ^1^ per Fruit	Fruit DW ^2^ per Fruit	Fruit Water Content ^3^
	Each	Brix	g	g	g	g	%
CTRL	3.93	14.03	568.2	109.0	144.4	27.71	81.0
	(0.81)	(0.314)	(121.5)	(22.69)	(5.514)	(1.270)	(0.38)
INOC	2.28	15.45	377.7	70.91	165.2	31.02	81.3
	(0.51)	(0.244)	(88.44)	(16.79)	(4.415)	(1.274)	(0.54)
*p*-value	0.098	< 0.001***	0.216	0.189	0.008**	0.070	0.549

^1^ FW, fresh weight of the samples. ^2^ DW, dry weight of the samples. ^3^ Calculated by (FW – DW)/FW of fruit.
